# Embedded Sensing System for Recognizing Citrus Flowers Using Cascaded Fusion YOLOv4-CF + FPGA

**DOI:** 10.3390/s22031255

**Published:** 2022-02-07

**Authors:** Shilei Lyu, Yawen Zhao, Ruiyao Li, Zhen Li, Renjie Fan, Qiafeng Li

**Affiliations:** 1 College of Electronic Engineering (College of Artificial Intelligence), South China Agricultural University, Guangzhou 510642, China; lvshilei@scau.edu.cn (S.L.); w844718456@163.com (Y.Z.); 20203162097@stu.scau.edu.cn (R.L.); aoaiouwen@foxmail.com (R.F.); 17688015023@163.com (Q.L.); 2 Guangdong Laboratory for Lingnan Modern Agriculture, Guangzhou 510642, China; 3 Pazhou Lab, Guangzhou 510330, China; 4 Division of Citrus Machinery, China Agriculture Research System of MOF and MARA, Guangzhou 510642, China

**Keywords:** florescence information monitoring, YOLOv4, cascade fusion, FPGA, embedded sensing system

## Abstract

Florescence information monitoring is essential for strengthening orchard management activities, such as flower thinning, fruit protection, and pest control. A lightweight object recognition model using cascade fusion YOLOv4-CF is proposed, which recognizes multi-type objects in their natural environments, such as citrus buds, citrus flowers, and gray mold. The proposed model has an excellent representation capability with an improved cascade fusion network and a multi-scale feature fusion block. Moreover, separable deep convolution blocks were employed to enhance object feature information and reduce model computation. Further, channel shuffling was used to address missing recognition in the dense distribution of object groups. Finally, an embedded sensing system for recognizing citrus flowers was designed by quantitatively applying the proposed YOLOv4-CF model to an FPGA platform. The mAP@.5 of citrus buds, citrus flowers, and gray mold obtained on the server using the proposed YOLOv4-CF model was 95.03%, and the model size of YOLOv4-CF + FPGA was 5.96 MB, which was 74.57% less than the YOLOv4-CF model. The FPGA side had a frame rate of 30 FPS; thus, the embedded sensing system could meet the demands of florescence information in real-time monitoring.

## 1. Introduction

Florescence information monitoring is the fundamental technical basis and key management chain for achieving a high-yield and high-quality orchard for strengthening orchard management activities, such as flower thinning, fruit protection, pest control, and yield prediction [1,2]. Researchers at home and abroad have recently proposed several solutions for accurately recognizing fruit flowers in their natural habitat. Zhao et al. [3] applied improved flower extraction feature pyramid networks to extract the local area of tomato flowers using the YOLOv3 model to accurately classify tomato flowers at different flowering stages. Wu et al. [4] proposed an improved YOLOv4 model combining channel pruning to recognize the varieties of apple flowers. Dorj et al. [5] adopted a color detection algorithm to identify citrus flowers in their natural habitat by combining them with a counting algorithm to achieve yield prediction on a server. Ambrozio et al. [6] and Sun et al. [7] applied semantic segmentation networks in various scenarios to automatically identify different fruit flowers, such as apples, pears, and peaches. Liu et al. [8] and Cui et al. [9] employed the K-means clustering image segmentation technique to achieve flower recognition of kiwifruit and strawberry, which have high requirements for experimental scenes and lighting conditions. Zheng et al. [10] proposed an object-recognition technique, which was applied to eggplant flower recognition using hybrid dilated and ordinary convolution. Further, Lin et al. [11] applied a deep faster R-CNN model to achieve strawberry flower recognition for the detection requirement of dense small-scale objects in a complex-structure background. Xiong et al. [12] applied a semantic segmentation model to achieve flower recognition in litchi. Deng et al. [13] applied an instance segmentation model to achieve citrus flower recognition.

Most related researchers are currently focused on improving the object recognition effect of orchard crops with different florescence [14,15,16]. However, the orchard working environment is complex and changeable. Citrus, for example, has different proportions of buds and flowers in the flowering season and is easily infected with gray mold in a low temperature and high humidity [17,18]. However, timely regulation is needed to avoid a negative impact on fruit quality and orchard yield. As such, research on recognizing bud, flower, and gray mold in a natural environment is in line with the application requirements of citrus florescence information monitoring.

Based on this, YOLOv4-CF, which is a lightweight object recognition model for citrus bud, flower, and gray mold, was proposed using the software and hardware codesign pattern. After transforming, quantizing, and compiling, it was deployed to a field programmable gate array (FPGA) [19,20,21], which has a high parallel computing capability that can accelerate convolution neural network operations. Based on cascade fusion YOLOv4-CF + FPGA, an embedded system was designed and implemented for the real-time and accurate identification of citrus flowers and gray mold. The system can facilitate flower thinning to protect citrus and indirectly forecast the production of citrus orchards during florescence. In addition, it provides an intelligent decision-making basis for disease prevention and control, allowing citrus orchard managers to prevent diseases at the earliest opportunity and improve citrus quality at the same time. The main contributions of this study are as follows:

1. A lightweight object recognition model using cascade fusion YOLOv4-CF is proposed, which recognizes multi-type objects in their natural environments, such as citrus buds, citrus flowers, and gray mold.
2. A method for deploying convolutional neural networks to an FPGA embedded sensing system is provided.
3. An embedded sensing system for recognizing citrus flowers is designed by quantitatively applying the proposed YOLOv4-CF model to an FPGA platform.

This study is outlined as follows: Section 2 shows the collection and processing of experimental data; Section 3 introduces the design of the YOLOV4-CF model for recognizing citrus flowers and gray mold; Section 4 presents the YOLOV4-CF model migration and deployment process of the FPGA embedded platform; Section 5 analyzes the experimental results in detail; Section 6 concludes the study.

## 2. Experimental Data and Processing Methods

**Figure 1:**
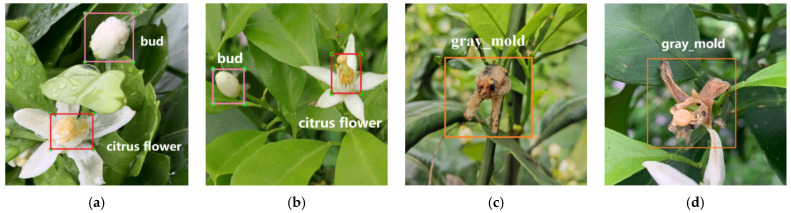
Sample labeling. (**a**) Citrus flower (rainy), (**b**) Citrus flower (sunny), (**c**) Gray mold (rainy), (**d**) Gray mold (sunny).

**Figure 2:**
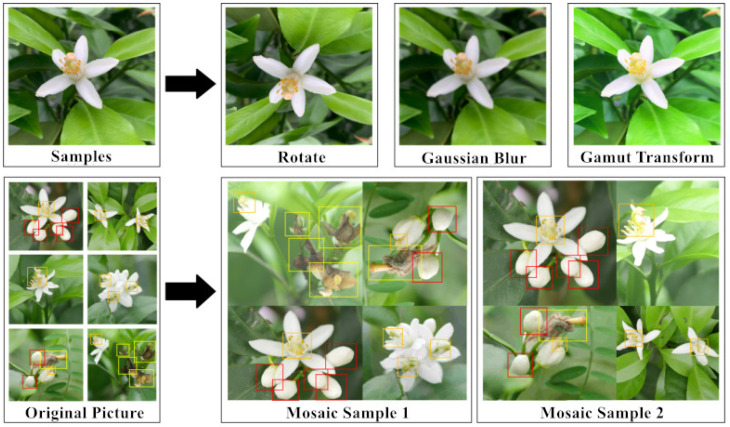
Mosaic data augmentation.

The data of citrus flowers and gray mold were collected in April 2021, and over 1000 fruit trees were collected from citrus orchards in Ziyuan County, Guilin, Guangxi, with the main variety of flower being Red Beauty (Ehime-ken 28). The fruit trees were captured from the four cardinal points (east, west, north, and south) using a Panasonic DMC-G7 camera and a high-definition mobile phone under natural environmental conditions (rainy and sunny days). Figure 1 shows that citrus bud, flower, and gray mold were tagged manually using the labelImg (version 1.8.3) software. The mosaic method [22] was employed to enhance the dataset in the training process of 1078 collected original pictures of citrus flowers (including buds and flowers) and gray mold. Each time, four pictures were randomly selected from the training data for flipping, Gaussian blurring, and color gamut transformation, among others, to randomly scale the pictures. Figure 2 shows that after randomly splicing into new pictures, they were sent to the training network for feature extraction, which enhances the object recognition model’s discrimination ability of similar features and improves the recognition accuracy. Table 1 shows a total of 1671 picture datasets after scaling, cropping, and flipping, which were divided into training sets and test sets with a ratio of 8:2.

## 3. YOLOv4-CF Accurate Recognition Model

**Figure 3:**
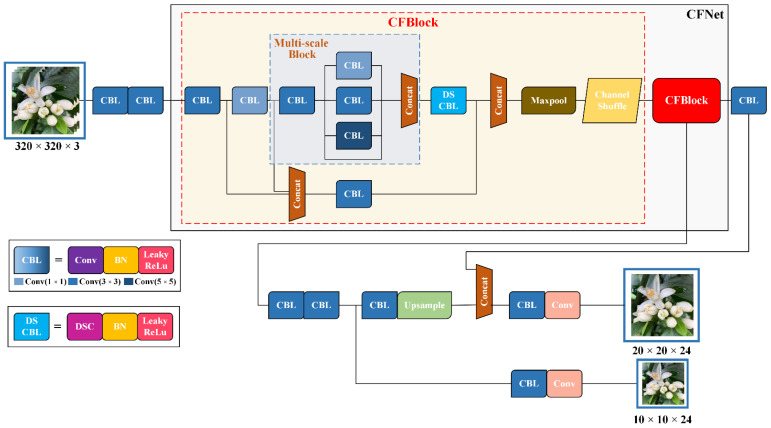
YOLOV4-CF neural network framework.

Citrus flower buds are difficult to accurately identify because they are densely distributed in their natural environment, and the characteristics of flowers in the early stage of gray mold infection are similar to those of normal flowers, which may lead to missed or false detection. Figure 3 shows a YOLOv4-CF (Cascade Fusion) lightweight object recognition model. The model is based on YOLOv4-Tiny [22,23] combined with Xception [24] and ShuffleNetv2 [25]. The improved cascade fusion network, CFNet, was used to replace CSPdarknet53-Tiny in YOLOv4-Tiny in the backbone feature extraction network. CFNet is primarily formed by stacking two cascade fusion blocks (CFBlock), which enhances the representation ability of the model without increasing the size by solving the problem of over-fitting and improving the recognition accuracy of small object groups with a dense distribution and similar features.

This study proposes the following three strategies for the improved CFBlock module: (1) a multi-scale block module designed to strengthen semantic information fusion at different levels. (2) To reduce recognition model computation, the traditional convolution block after the multi-scale feature fusion module is replaced by a depth separable convolution block. (3) Channel shuffle operation [26] is added to the CFBlock module to reorganize different features of the object to improve the interaction among features. Further, these strategies are summarized as follows:

1. Multi-Scale Block

**Figure 4:**
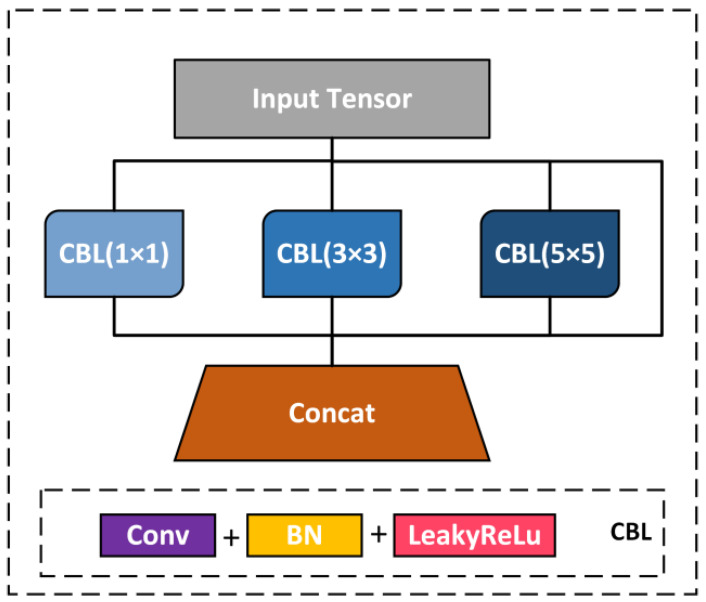
Multi-scale block framework.

Figure 4 shows that a multi-scale feature fusion layer module is designed to improve the information interaction among multiple levels in the recognition model and enhance the model’s recognition accuracy for confusing objects.

The CBL module comprises three different modules with a 1 × 1 step size and 1 × 1, 3 × 3, and 5 × 5 convolution kernel sizes. Each CBL module comprises ordinary convolution, batch normalization (BN), and LeakyReLu activation function. Continuous feature transformation increases the expression dimension of features and facilitates the fusion of semantic information at various levels. The concatenate operation was chosen to connect the three CBL modules to preserve as much spatial information as possible. The samples on the feature maps of different scales are displayed to the same size for fusion, whereas the number of channels remained constant; it expands the dimension of the tensor and reduces the loss of information by improving the adaptability of the whole network to scale and making the output feature information richer.

2. Depth Separable Convolution Block

To further minimize the size and computation of the recognition model and combine the properties of the Mobilenet model [27,28], the traditional convolution block after replacing the multi-scale feature fusion module with the depth separable convolution layer helps reduce the parameter quantity compared with the conventional convolution operation, which benefits the network’s light weight.

If the size of the input feature graph is *D_f_* × *D_f_*, number of channels is *M*, and the size of the convolution kernel is *D_k_* × *D_k_*; the traditional convolution operation is *D_f_ × D_f_ × M × N × D_k_ × D_k_*, whereas the deep separable convolution operation is *D_f_ × D_f_ × M × D_k_ × D_k_ + D_f_ × D_f_ × M × N*, and the ratio of the traditional convolution operation is $\frac{1}{N}\;$*+*$\;\frac{1}{D_{k\;}\times \;D_{k}}$. Thus, the computational complexity of the depth separable convolution is directly proportional to the output channel *N* and convolution kernel size *D_k_* × *D_k_*. The YOLOv4-CF model uses a 3 × 3 convolution kernel with 64 and 128 output channels, and the depth separable convolution has a computational complexity of 0.127 and 0.119 times that of typical convolution parameters, which significantly accelerates the speed model’s reasoning.

3. Channel Shuffle

The enhanced CFBlock module proposed herein uses concat to connect the feature map information features of various branches and adds channel shuffling operation to identify information after concatenation and fusion, transmitting it down to solve the problem of information non-circulation between upper and lower modules. Further, the information interaction among features is improved during transmission to ensure that the information fusion among channels is completed without increasing the amount of computation and parameters, so the image features of each part can be effectively transmitted to the next module to enhance the ability of model feature extraction.

## 4. FPGA Embedded Platform and Recognition Model Migration and Deployment

The FPGA embedded platform used herein is the Zynq UltraScale + MPSoC ZCU104 (Produced by Xilinx Company, Singapore) operation platform [29], and it includes FPGA and ARM processing systems, a 504 K system logic unit, a quad-core ARM Cortex A53 application processor, a dual-core Cortex-R5 real-time processor, and a deep learning processor unit (DPU), which supports different neural network structures through reconfigurable hardware architecture.

**Figure 5:**
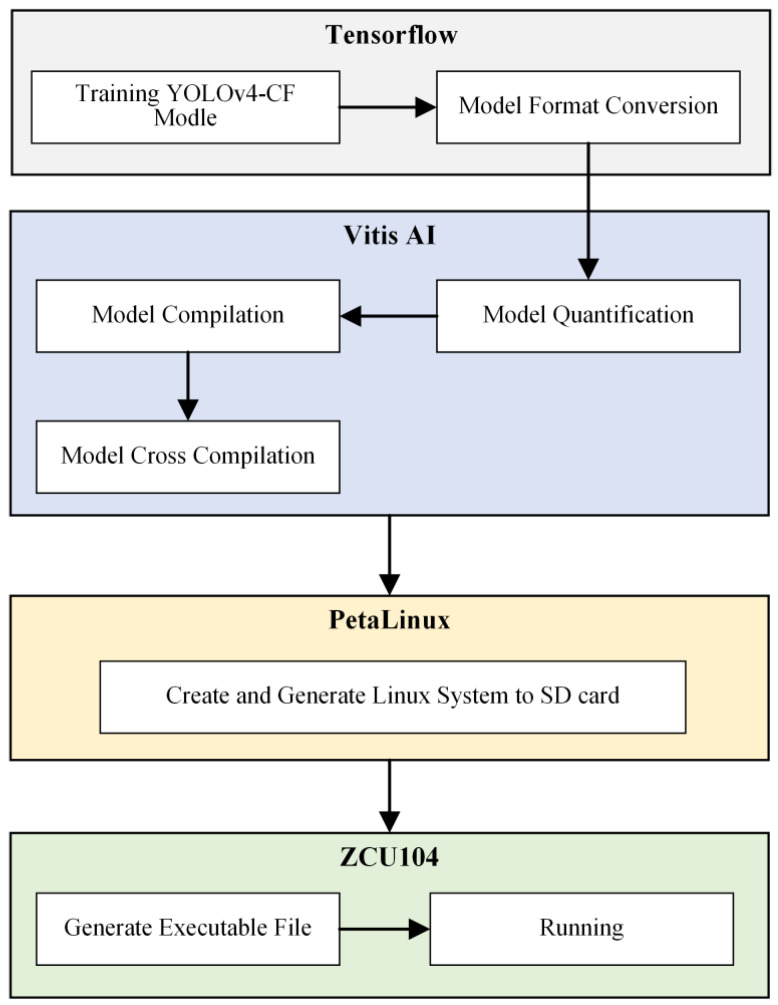
YOLOv4-CF + FPGA object recognition embedded system process.

Figure 5 shows the workflow chart of the YOLOv4-CF + FPGA object recognition embedded system. To achieve a real-time processing system of video capture data, a USB, Micro SD, DDR, DisplayPort, and UART interface for debugging should be connected. The ARM processing system (PS) and FPGA programmable logic (PL) are the two components of ZCU104, both of which communicate through the AXI bus. The processing system is primarily responsible for acquiring, storing, reading, scaling format converting, and displaying the processing results of the object image; it uses DPU to accelerate the processing of the YOLOv4-CF neural network object recognition model in FPGA.

**Figure 6:**
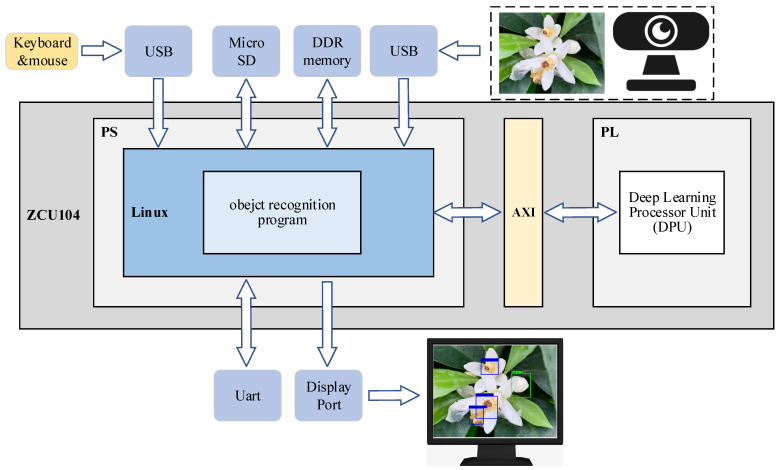
YOLOv4-CF + FPGA object recognition model deployment process.

Figure 6 shows the main steps of the YOLOv4-CF + FPGA object recognition model migration and deployment process, which comprise three parts: model format conversion in a TensorFlow environment on the server, the compilation and quantification of a model in a Vitis AI environment, and programming processing of the ZCU104 platform on the embedded FPGA side. In the first part, the Xilinx Vitis AI tool is affected by the technical update iteration speed and version limitation [30] because it does not support the direct quantification of Keras weight files created in the TensorFlow environment. Thus, this study transforms the weight of the Keras model and network architecture file into a binary protobuf file. Model freezing, node pruning, and other procedures are performed to remove the network nodes that do not affect the output tensor during the transformation process. In the second part, the structure of FPGA determines whether it can perform a fast floating-point operation; thus, this study converts the 32-bit floating-point number of the trained citrus flower recognition model into an 8-bit integer fixed-point number. Further, the citrus flower training set is used as the calibration dataset. The recognition model is quantized at a fixed point using the Vitis AI quantizer tool, which is combined with the converted protobuf file. Based on the FPGA embedded platform model, this study uses the B4096 convolution structure to deploy the algorithm model and accelerates the use of the Xilinx AI compiler to build the quantized model. The depth neural network compiler (DNNC) outputs the elf file and DPU kernel information required by the DPU. Finally, the cross-compile tool is used to process the elf file generated by the Vitis AI tool to build a Linux dynamic link library file to be transplanted into the ZCU104 embedded platform. The PetaLinux tool is used to build the embedded Linux operating system in the last part. To process the image at ARM for the Python program, OpenCV library, DPU library functions, and API are used; DPU is employed to achieve the real-time recognition of citrus flowers.

## 5. Results and Analysis

To evaluate the performance of the embedded system for the accurate recognition of citrus flowers and gray mold, combined with various improvement strategies of the YOLOv4-CF model, object recognition performance tests are performed based on various test platforms, such as the server, PC, and FPGA. Table 2 shows the configuration environment of each test end. As shown in Equations (1)–(4), this study uses the mean average precision (mAP@.5) and *F*1 measure as the evaluation indexes of the YOLOv4-CF model for multi-object detection precision. Further, FPS is employed as the evaluation index of the YOLOv4-CF + FPGA model detection speed.

(1) $$P=\frac{T_{P}}{T_{P}+F_{P}}\times 100\%$$

(2) $$R=\frac{T_{P}}{T_{P}+F_{N}}\times 100\%$$

(3) $$F1=\frac{2PR}{P+R}\times 100\%$$

(4) $$mAP=\overset{1}{\underset{0}{\int }}P\left(R\right)dR\times 100\%.$$

where *P* represents the precision rate, *R* the recall rate, *T_P_* the number of true positive samples, *F_P_* the number of false-positive samples, and *F_N_* the number of false-negative samples.

### 5.1. Analysis of the YOLOv4-CF Model Training Results

**Figure 7:**
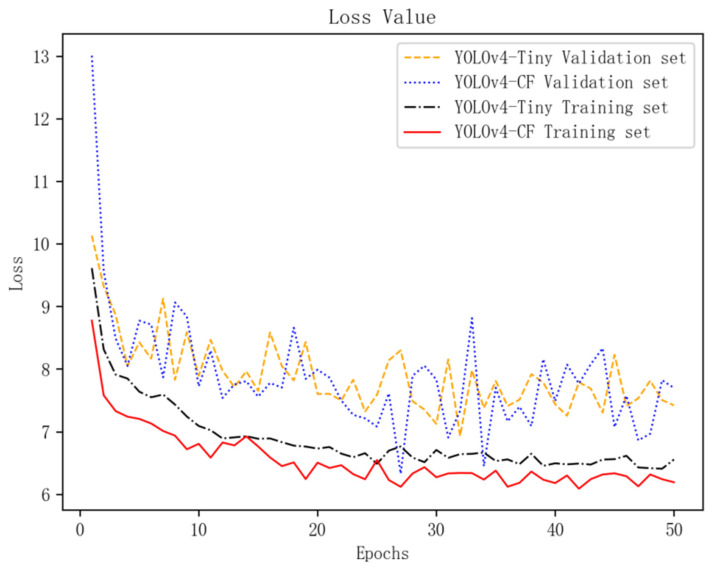
Training loss value of the two models.

This study performs 50 epochs training on the YOLOv4-CF and YOLOv4-Tiny models based on the same dataset and training parameters and then employs the model results of the previous training as the pretraining model for the transfer training to evaluate the training performance of the model. The training to verification set is 8:2; the training process has a reverse gradient, whereas the verification process does not. Further, the YOLOv4 standard loss function is used as the model training performance index, as shown in Equation (5). Figure 7 shows the loss curves of each model after migration training. Moreover, location_loss uses CIoU loss as location loss.

(5) Loss=location_loss + confidence_loss + class_loss

Figure 7 shows the training loss value of the YOLOv4-CF and YOLOv4-Tiny models fitted quickly in the first 10 iterations, with the loss value dropping rapidly and then decreasing slowly. In the early stage of training, the YOLOv4-CF model’s validation set loss is higher than that of the YOLOv4-Tiny model; the overall fluctuation range is larger, but the convergence speed is faster. The training set loss value of the YOLOv4-CF model is lower than the YOLOv4-Tiny model with a better fitting degree, indicating that the YOLOv4-CF model has a superior convergence effect. Finally, the training set’s loss value is stable between 6.0 and 7.0, with only a slight oscillation, indicating that model fitting is over.

### 5.2. Performance Analysis of the Improved Strategy of the YOLOv4-CF Model

**Figure 8:**
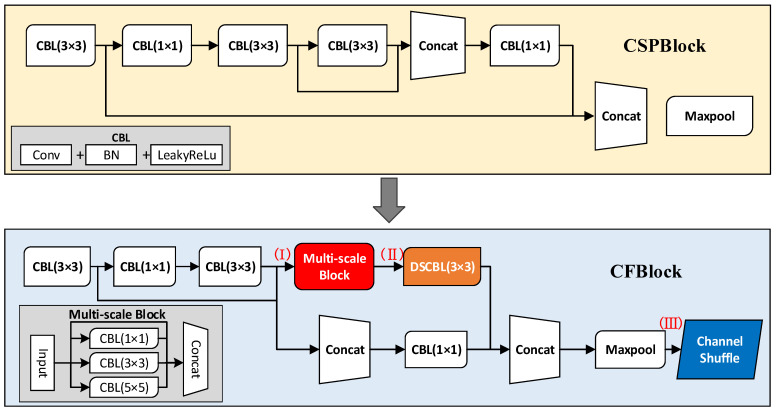
Improved structure comparison.

To further analyze the performance of various improvement strategies, based on the same training sets and test environments, this study effectively tests the performances of various improved strategy combinations of the YOLOv4-Tiny and YOLOv4-CF models. The three residual modules CSPBlock in the original YOLOv4-Tiny network are pruned into two. This study further modifies its structure and designs a multi-scale block to form the YOLOv4-CF (I) model. Based on this, the ordinary convolution after the multi-scale block is replaced by depth separable convolution to form the YOLOv4-CF (II) model. Finally, the channel mixing operation is added to form the YOLOv4-CF model. Figure 8 shows the residual module CSPBlock in YOLOv4-Tiny and the improved cascade fusion module CSPBlock in YOLOv4-CF. To simultaneously improve the model recognition effect based on the relatively fixed aspect ratio of citrus bud, flower, and gray mold in the dataset, a K-means clustering algorithm [31] is employed to optimize the object clustering center and obtain the following six groups of anchor boxes that meet the object size: (18, 22), (25, 34), (35, 42), (43, 58), (68, 78), and (116, 136). Further, this study uses 320 × 320 as the training set input size. Two models are initially trained using 50 epochs, and then the model results from the previous training are used as the pretraining model for migration training. Table 3 shows the test results of different models in the test set.

Table 3 shows that three improved strategies such as multi-scale block, depth separable convolution block, and channel shuffling operation contribute to improving the recognition precision of the YOLOv4-CF model. Although the precision of flower in YOLOv4-CF(I) model is slightly lower than that of YOLOv4-Tiny in the test results of the initial training, the average precision of citrus bud and gray mold is 91.33% and 92.94%, which were increased by 1.23% and 3.78%, respectively. The precision of gray mold has also been greatly improved, and the *F*1 value was increased by 2%, which indicates that the cascade fusion model with the multi-scale block strategy has better representation ability, and the features of citrus bud and gray mold can be better extracted by the improved model. The deep separable convolution is added to the YOLOv4-CF(II) model. The average precision mAP@.5 value is increased by 2.58% compared to YOLOv4-Tiny, and the recognition precision of the citrus flower, citrus bud, and gray mold is improved, solving the problem of the decreasing recognition precision of citrus flowers. Gray mold’s recognition precision is 94.65%, which is 5.49% higher than that of the original YOLOv4-Tiny model, and 1.71% higher than that of the YOLOv4-CF(I) model with an increased parameter of only 0.1 MB, indicating that adding deep separable convolution can ensure that the recognition precision of the model can be significantly improved while the model parameters are constant. The YOLOv4-CF model, which is based on the YOLOv4-CF (II) model, adds channel mixing operation, and its average precision mAP@.5 value reaches 95.42%, which is 3.09% higher than that of the YOLOv4-Tiny model. Further, the AP values of the three recognition objects and the *F*1 values of the model are improved. Compared with the YOLOv4-CF(II) model, the *F*1 value and model parameters remained constant, but mAP@.5 increased by 0.51%. In summary, the model with the channel mixing operation has higher feature fusion ability and significant advantages over the initial YOLOv4-Tiny model.

After using transfer learning in the test results of the second training while keeping the parameters constant, the recognition precision of all models is improved compared with that of the first training. Further, the various indexes of the YOLOv4-CF model after initial training are higher than those of the YOLOv4-Tiny model after transfer learning, indicating that the optimized YOLOv4-CF model has higher recognition precision. The results of the transfer learning training demonstrate that the precision mAP@.5 value of the YOLOv4-CF model is 95.03%, and the *F*1 value is 89.00%, which is 1.42% higher than that of the YOLOv4-Tiny model, and the recognition precision of the citrus flower, citrus bud, and gray mold is improved by 0.94%, 0.72, and 2.07%, respectively. As the identification precision of gray mold significantly improved, the *F*1 value increased by 3%, and the parameter quantity increased by only 0.7 MB. The recognition model uses the stamens of citrus flowers as the recognition object because its color and shape features are more evident than the other two types of recognition objects; thus, the recognition precision is the highest, and the AP is 96.22%. However, citrus buds are thick with small volumes, and their color is similar to that of blooming citrus petals, which are easily misidentified or missed by the model. Thus, its precision is the lowest, with an AP of 92.89% among the three types of recognition objects.

In addition, we added ten accuracy comparison tests, each of which randomly selected 200 pictures from the citrus flower dataset as the test set. The trained YOLOv4-CF and YOLOv4-Tiny models were evaluated under the same operating environment, and ten sets of data were obtained for paired sample *T*-test. According to the test result, the significant difference *p* = 0.000032 is less than 0.001, indicating that YOLOv4-CF and YOLOv4-Tiny models have significant differences.

To further prove the performance of the YOLOv4-CF model, the feature extraction network of YOLOv4-Tiny was replaced with different lightweight convolutional neural networks for comparative experiments. The experiment used the same training and test sets for model training and accuracy testing in TensorFlow(GPU). Speed tests were performed on the same picture with input sizes of 460 × 680. The convolutional neural networks compared were MobileNet-V3, GhostNet, DensNet121, CSPdarknet53_tiny, and CFNet. Furthermore, CSPdarknet53_tiny is the feature extraction network of YOLOv4-Tiny and the CFNet is the feature extraction network of YOLOv4-CF. The experimental results are shown in Table 4.

From the empirical observations, it can be noted that CFNet has many distinct benefits over other feature extraction networks. The mAP and speed of testing a picture are 95.03% and 17.93 ms, respectively, which are better than other models. Moreover, when GhostNet is used as the feature extraction network, the model size is the smallest. However, its picture test speed is 27.42 ms, which is 9.49 ms slower than CFNet. The mAP is 92.74% when the model’s feature extraction network is DensNet121. However, the model size and the speed are the largest and slowest, which does not meet the requirements of edge platforms. In addition, when the feature extraction network of the model is CSPDarkNet53-Tiny, the metrics are the closest to the results of CFNet, but the test speed is slower than that of CFNet. This proves that the model complexity of CFNet is lower, and it is more in line with the requirements of embedded platform transplantation. In summary, CFNet as a feature extraction network has the best comprehensive performance and the most robust feature extraction ability.

**Figure 9:**
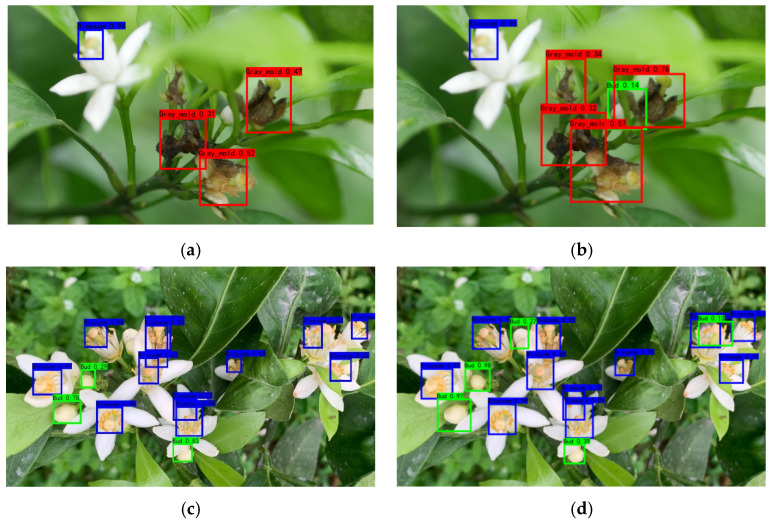
Test results of the YOLOv4-CF model and the YOLOv4-Tiny model on citrus flowers in the natural environment. (**a**) Sparse object (YOLOv4-Tiny), (**b**) Sparse object (YOLOv4-CF), (**c**) Intensive object (YOLOv4-Tiny), (**d**) Intensive object (YOLOv4-CF).

Figure 9 shows the YOLOv4-CF model and YOLOv4-Tiny model used to test citrus flowers in the natural environment. The recognition frames of citrus bud, citrus flower, and gray mold are set as green, blue, and red, respectively. The test results of citrus flowers with sparse and intensive objects show that multiple types of objects that YOLOv4-CF correctly recognize almost all the targets in the figure, whereas YOLOv4-Tiny has more omitted detections and false detections. The comparison results show that YOLOv4-CF has higher accuracy and a better recognition effect on gray mold.

### 5.3. Resulting Analysis of the YOLOv4-CF + FPGA Model

As described in Chapter 4, the ZCU104 computing platform is used as the migration deployment test platform, with a size of 150 mm × 180 mm × 50 mm, which weighs approximately 500 g with rich computing resources that meet the application requirements of citrus flower object real-time recognition embedded systems. The YOLOv4-CF model is quantized and developed on the PC and transplanted into the ZCU104 platform to form the YOLOv4-CF + FPGA model. Further, this study uses a 640 × 480 dataset that successively tests the performance of the recognition model on the server (CPU and GPU) and FPGA. Table 4 shows the test results.

**Figure 10:**
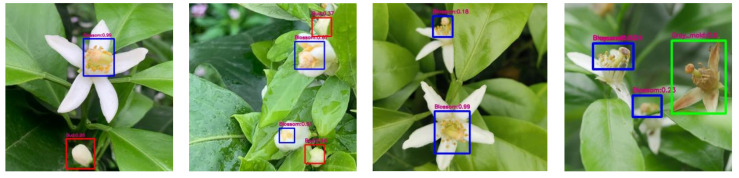
YOLOv4-CF + FPGA model recognition results of citrus flowers.

Table 5 shows that GPU has the best recognition precision of gray mold on citrus flowers with up to 96.20%, and the average recognition precision on the server is 94.11%. The average recognition precision on the embedded platform is 92.83%, the recognition model’s precision loss after quantitative transplantation is 1.28%, and the recognition speed is 58.74 ms, which is slower than the GPU accelerated recognition speed, but faster than the CPU recognition speed. The size of the recognition model is 5.96 MB, with a reduction of 74.57%. The quantization tool needs to perform fixed-point quantization on the weights and activation values of the entire model. As a result of this procedure, the model’s detection accuracy will be reduced to achieve faster computation speeds and a greater compression ratio. Figure 10 shows the recognition results of citrus flowers in various scenes using the YOLOv4-CF + FPGA model. To distinguish between the server recognition results, the recognition boxes of citrus bud, citrus flowers, and gray mold are set to red, blue, and green, respectively. In summary, the citrus flower accurate recognition system can effectively enhance object recognition precision and recognition speed through small model precision loss and tackle the portability and real-time issues of citrus orchard information monitoring.

**Figure 11:**
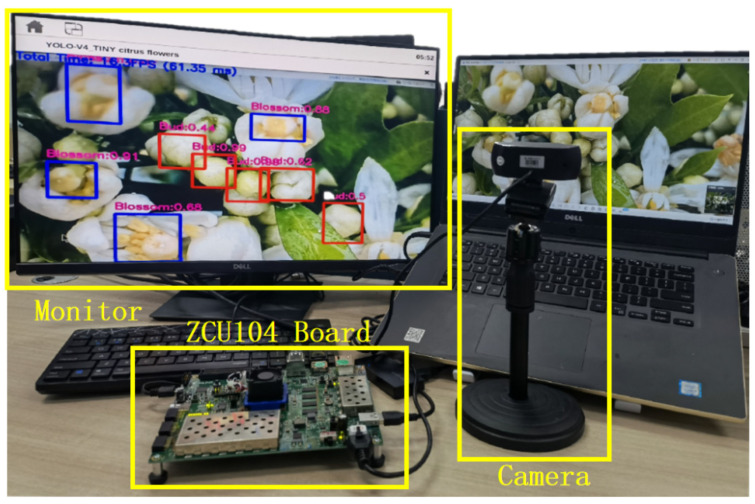
FPGA video stream test scheme.

To verify the real-time recognition efficiency of the citrus flower accurate recognition system, this study compares and tests the recognition effects of server, PC, and FPGA on a video stream. The video stream resolution is 640 × 480, and the power metering socket is used to monitor the power consumption of various test platforms. Table 5 shows the test results. Figure 11 shows the FPGA video stream test scheme to restore the actual application scenario of the embedded recognition system simultaneously. The camera is connected to ZCU104 through a USB interface and records the PC’s screen video stream in real time; the display is connected to ZCU104 through the DP interface, which displays the recognition results processed by ZCU104 in real time.

Table 6 shows that the server RTX 2060GPU camera has the fastest real-time recognition speed of 29 FPS, but the total power consumption is 96 W, which is five times more than the FPGA embedded platform; the consumption efficiency of the FPGA embedded platform is 20 W. Compared with the MX150 GPU on the notebook computer and core i7-9700 CPU on the server, the power consumption is reduced by 46 and 55 W, respectively. The recognition speed of the FPGA embedded platform is 17 FPS, which is only slower than the server RTX 2060 GPU. It has a noticeable advantage in speed and power consumption compared with other platforms. To further test the real-time speed of the FPGA embedded platform camera, different input sizes are compared. The results show that the input size is 640 × 480, recognition speed is 17 FPS. However, if the input image size is 320 × 240, recognition speed can clock 32 FPS, approximately twice as fast as the 640 × 480 input size. In summary, different input image sizes are suitable for real-time scenes with different requirements.

## 6. Conclusions

A citrus flower recognition method based on an improved YOLOv4-CF lightweight neural network was proposed herein, and the cascade fusion network, CFNet, was introduced to replace the original backbone network. The experimental results showed that, in the natural environment, the mAP@.5 and *F*1 values of the server YOLOv4-CF model achieved 95.03% and 89.00%, respectively; this is an increase of 0.94% and 1.41%, respectively, compared with the YOLOv4-Tiny model, which provides reliable data support for orchard flowering information monitoring.

This study uses model deployment to design a citrus flower accurate recognition system based on an FPGA embedded platform. The size of the system model after quantitative compilation is 5.96 MB, which is only 25.47% of the original model; the recognition precision is 92.83%, and the power consumption is 20 W, which is only 20.8% of the GPU power consumption. Recognition speed is approximately 30 FPS if the input size is 320 × 240. It has the characteristics of low energy consumption and easy portability, which meets the requirements of real-time recognition of citrus flowers.

Further work is needed to improve the proposed method. For example, FPGA computing resources were not fully used, and the system’s real-time performance can be improved. In the next research project, the author will expand the dataset and enhance the field experiments in citrus orchards. Further, the PL should be connected to the MIPI camera at the embedded FPGA to replace the USB camera; this is to improve the stability and the system’s real-time performance by fully using the computing advantages of FPGA.

## Figures and Tables

**Table 1:** Datasets of citrus flowers and gray mold.

Tag Name	Training Set	Test Set	Total
bud	1005	249	1254
citrus flower	1223	315	1538
gray mold	615	141	765

**Table 2:** Configuration of different test equipment.

No.	Platform	System	Configuration	Operating Environment
1	Server	Windows 10	Intel Core i7-9700 @ 3.00 GHz Eight-core CPU, 16 GB RAM, Nvidia GeForce RTX 2060(6 GB) GPU	The test framework is TensorFlow 2.2.0, Keras 2.3.1, using CUDA 10.1 parallel computing framework with CUDNN 7.6.5 deep neural network acceleration library
2	PC	Windows 10	Intel Core i7-8500U @ 1.80 GHz Four-core CPU, 16 GB RAM, Nvidia GeForce MX 450(4 GB) GPU	
3	FPGA	Linux	Xilinx Zynq UltraScale + MPSoC EV (ZCU104) SoC	Compile environment uses OpenCV and Xilinx AI runtimes

**Table 3:** Test results of different models.

Traning	Model	mAP@.5%	AP/%			*F*1%	Model Size/MB
			Bud	Flower	Gray Mold		
Initial training	YOLOv4-Tiny	91.33	90.10	94.75	89.16	82.67	22.79
	YOLOv4-CF(I)	92.94	91.33	94.56	92.94	84.67	23.31
	YOLOv4-CF(II)	93.91	91.76	95.32	94.65	86.33	23.44
	YOLOv4-CF	94.42	92.17	95.67	95.42	86.33	23.44
Transfer training	YOLOv4-Tiny	93.61	92.17	95.28	93.39	86.00	22.79
	YOLOv4-CF(I)	93.61	92.20	95.22	93.42	86.67	23.31
	YOLOv4-CF(II)	94.36	92.05	95.57	95.46	88.00	23.44
	YOLOv4-CF	95.03	92.89	96.22	95.97	89.00	23.44

**Table 4:** Performance of different methods.

Method	mAP (%)	Test Time (ms)	Model Size (M)
MobileNet-v3 [32]	85.76	23.93	18.43
GhostNet [33]	88.63	27.42	11.50
DensNet121 [34]	92.74	42.12	39.11
CSPDarkNet53_Tiny [22]	94.42	18.83	22.79
CFNet (ours)	95.03	17.93	23.44

**Table 5:** Recognition results of different platform test sets.

Platform	Precision/%			Average Precision /%	Model Size /MB	Recognition Speed/ms	
	Bud	Flower	Gray Mold				
Server	91.47	94.65	96.20	94.11	23.44	69.22 (CPU)	33.28 (GPU)
FPGA	89.22	93.54	95.73	92.83	5.96	58.74	

**Table 6:** Real-time recognition efficiency of different platforms.

Platform	Configuration	Speed/FPS	Power/W
Server	CPU/core i7-9700(3.0 GHz Eight-core)	15	75
	GPU/RTX 2060(6 GB)	29	96
PC	CPU/core i7-8550U(1.8 GHz Quad-core)	10	38
	GPU/MX150(4 GB)	16	56
FPGA	ZCU104	17	20

## Data Availability

Not applicable.
